# Repurposing research data for commercial use: POPIA, a foil or a facilitator?

**DOI:** 10.17159/sajs.2023/15075

**Published:** 2023

**Authors:** Beverley Townsend, Amy Gooden, Marietjie Botes, Donrich Thaldar

**Affiliations:** 1 York Law School, University of York, York, UK; 2 School of Law, University of KwaZulu-Natal, Durban, South Africa; 3 SnT Interdisciplinary Centre for Security, Reliability, and Trust, University of Luxembourg, Luxembourg, Luxembourg; 4 Petrie-Flom Center for Health Policy, Biotechnology, and Bioethics, Harvard Law School, Cambridge, Massachusetts, USA

**Keywords:** research, commercialisation, repurposing, POPIA, Draft Code of Conduct for Research

## Introduction

It is common practice for institutions around the world to conduct research using donated biological samples and related data. But what if a research institution wants to use the data generated from these samples for commercial applications, or license the use of the data, or sell the data to a commercial entity? Allegations of the commercialisation of data initially collected for research are not new, with companies, universities, and state departments purportedly selling and commercialising biological samples and/or the data originally collected for research purposes.^1–7^ It is not only a matter of the repurposing of data initially collected for research for commercial use, but crucially that these activities may have been done without the knowledge or consent of the participants to whom the data relate. Individuals, it would seem, are less likely to donate altruistically if, unbeknown to them and without their consent, an organisation is to benefit financially from their contribution.^7^

Sound data protection practices form the backbone of lawfully and successfully securing public confidence in data use.^8^ This is particularly true in light of the dramatic increase in the volume of digitally available research information coupled with the ability to share such data widely. To this end, the South African *Protection of Personal Information Act 4 of 2013* (POPIA) sets out measures to safeguard personal information. While much of the literature has focused on data collected for a commercial purpose – such as data stored in a commercial biobank – being further processed (or ‘repurposed’) for research use^9–18^, we consider here the implications when the position is reversed: that is, when data collected for research are repurposed for commercial use. This would typically be the case if research yields data that are commercially valuable. Examples would be data sets containing the genomic sequences and health information of large groups of people.

Our intention in this Commentary is threefold. First, we suggest that the commercialisation of research data is a significant issue for the South African research community, as it is not only a public policy imperative, but, in certain crucial circumstances, a statutory duty.

Second, and in light of this, we analyse how POPIA might act both as a foil and a facilitator of secondary data use for purposes of commercialisation. This is to say that we consider, first and foremost, the legal position regarding the repurposing and commercialisation of research data, as stipulated in POPIA, rather than taking any ethical position one way or the other. We acknowledge that compelling ethical reasons underlie the requirement for, and availability of, high-quality, curated, unbiased, and representative data, and that there is enormous value and utility in granting wider access to discrete, aggregated data sets. Equally, however, and precipitated on important value judgements involving autonomy and self-determination, personal data may be used beyond the context for which consent was initially granted or ‘repurposed’ for other uses where the downstream commercial applications cannot always be anticipated, often contrary to user expectations. For example, the unsupervised and unregulated mass collection of biometric data was recently reported in 23 African countries by various third-party actors, the use of which is for unknown purposes.^19^ These are, unfortunately, not isolated anomalies, but are symptomatic of troubling precedent-setting trends in countries with inadequate privacy legislation and enforcement, and where privacy protection is not taken seriously. Guided by values, virtues, and considerations of human well-being, we need to rethink not just how data sets are used and re-used and for what purpose, and how innovative applications are built, but for whom and at what cost. Here, we thus demonstrate only, and narrowly, how POPIA helps to set guardrails for the repurposing of data for commercial use through the deployment of lawful data practices.

Finally, we make recommendations on how the proposed POPIA draft Code of Conduct for Research (for ease, we refer to it simply as the ‘draft CCR’), recently published by the Academy of Science of South Africa (ASSAf)^20^, can support the legislative framework by guiding the South African research community in this regard.

## The importance of commercialising research data

Research and research data are invaluable to innovation and contribute significantly to the advancement of society. Research, while primarily and traditionally the domain of university research groups and university hospitals, is now increasingly being conducted by various actors and is frequently used in commercial applications.^21^ Universities remain one of the primary institutions driven to commercialise research. Investment in emerging research areas leads to better funding, new jobs, industry development, and enhanced collaboration between universities and industry, which is often required in translating research into valuable products and therapies for the public.^22,23^ Examples of such applications are in producing vaccines^24,25^, in clinical trials^26,27^, and in the development of medications, treatments, and products by pharmaceutical companies, and others^22,26–29^. Innovation and commercialisation promote economic growth and, in turn, advance human well-being and benefit society.^23^

South African science and innovation policy supports the commercialisation of research results.^30–33^ This public policy position of support for commercialisation has even been enacted as a *statutory duty* in certain circumstances. The *Intellectual Property Rights from Publicly Financed Research and Development Act 51 of 2008* (IPR Act) provides that where publicly financed research results in intellectual property (IP), such IP *must* be protected and commercialised in a way that benefits South Africa (section 2 of the IPR Act). In other words, from a South African public policy viewpoint, research is not (merely) a purpose in itself, but has the instrumental goal of building the South African economy. This public policy perspective of research underpins our analysis in this article.

## Analysis

### Preliminary observations

Concerning commercialisation, there are two scenarios at play: first, consideration of the repurposing of data, initially collected for a commercial purpose, for a subsequent research purpose; and second, consideration of the inverse position, that is, where data initially collected for research are commercialised. The tangential question of whether research participants are entitled to the benefits of such commercialisation is beyond the scope of this article. We provide two points of clarification before the position regarding purpose specification and further processing is described.

First, POPIA does not apply to the processing of de-identified personal information that is incapable of being re-identified (section 6(1)(*b*)), hence research data that have been de-identified and then used for commercial purposes fall beyond the remit of POPIA. True de-identification of data (and because of their nature, genomic data) is questionable.^9,34–37^ Such data fall within POPIA, unless de-identified or otherwise excluded in terms of section 6.

Second, the *Regulations Relating to Research with Human Participants* (Human Research Participant Regulations)^38^, published in terms of the *National Health Act 61 of 2003* (NHA), provide that research participants must be informed of, inter alia, ‘the expected benefits of the research’ (regulation 5(*e*)) and ‘the availability of beneficial products or interventions post-research (regulation 5(*n*)) – the implications of which are that any possible commercialisation must be communicated to research participants.

### Purpose specification and further processing limitation

At the outset, section 13(1) of POPIA requires that personal information ‘be *collected for a specific, explicitly defined and lawful purpose*’ that is ‘*related to a function or activity of the responsible party*’ and that (except for cases that fall within section 18(4) of POPIA) the data subject (the individual ‘to whom personal information relates’ (section 1 of POPIA) which, in this case, is the research participant) must *be made aware* of the purpose of the collection of the personal information (section 13(2)). Therefore, where data that are initially collected for the specific and explicitly defined purpose of research are subsequently re-used for a different purpose, such as selling the data, and reasonably practicable steps are not taken to make the data subject aware of this, this contravenes section 13(2) of POPIA.

However, following this, and taking into account the specific purpose for which the data were collected (referred to in section 13 of POPIA), any further processing of personal information must be in accordance with (or compatible with) this specific purpose (section 15(1) of POPIA). So, in the event that the data were collected for a research purpose, any subsequent research would be allowable as it is in accordance with its initial purpose, that is, of research. However, on a reading of sections 13 and 15 of POPIA, *prima facie*, this means that where the activities of the responsible party do not include commercial purposes, and the data were not collected for a commercial purpose (that is, the purpose was solely for research), then the responsible party is not permitted to further process this information for a commercial purpose as it falls outside the scope of their activities and is not compatible with that initial purpose – namely research.

### POPIA: ‘Compatible’ purpose

POPIA sets out what is meant by ‘compatible’ and what are deemed ‘not incompatible’ purposes in section 15. Others in the literature have posed antithetical meanings of ‘compatibility’ as situated within research: whether an initial collection of personal information for medical research may be more broadly interpreted to mean that *any* other medically related research is compatible, or whether compatibility should be attributed a far narrower meaning – that is to say, data collected for medical research on a specific medical condition such as, diabetes, for example, would exclude medical research on other conditions, say asthma.^39^ We do not argue the particularities of these positions here, save to say that we examine the position regarding the ‘compatibility’ of data collected for research being repurposed for ‘commercial use’ – a use that we interpret in an extended sense as *any* commercial use and *any further* commercial use.

As a point of departure, section 15(2)(*a*)–(*e*) of POPIA implores us to assess the following criteria when determining compatibility: the relationship between the purpose of the further processing and collection (section 15(2)(*a*)), the nature of the information (section 15(2)(*b*)), the effect that the further processing may have on the data subject (section 15(2)(*c*)), the manner in which the information was collected (section 15(2)(*d*)), and any contractual duties that exist between the parties (section 15(2)(*e*)). Thus, to determine whether further processing is permitted in a given situation will depend upon the circumstances of the case, the conditions and contracts in place, as well as the information’s nature and effect, and its intended use.

Where data were collected for a non-commercial purpose, such as for research, the presupposition is that the data cannot be commercialised, as it would fall outside of the scope of the original purpose of collection, thereby precluding further processing for commercial purposes (in terms of section 15(2)(*a*) of POPIA). But all is not lost. Notwithstanding the section 15(2) compatibility criteria, POPIA lists instances where an otherwise incompatible purpose may be deemed compatible.

### POPIA: ‘Not incompatible’ purpose

POPIA clarifies specific instances, in section 15(3), that are deemed *not in*compatible (that is, they *are* compatible) with the purpose of collection. These instances are when: *consent* has been granted for the further processing (section 15(3)(*a*)), the information is contained in a *public record* or the data subject has *deliberately made it public* (section 15(3)(*b*)), the information is needed for *legal purposes or national security interests* (section 15(3)(*c*)), further processing is required in order to *prevent a threat to health or safety* (section 15(3)(*d*)), the information is used for ‘*historical, statistical or research purposes*’ and is further processed only for those purposes and will not be published in an identifiable manner (section 15(3)(*e*)), or an *exemption has been granted in terms of section 37* (section 15(3)(*f*)).

In light of the above, consider a situation in which data that were initially collected for a commercial purpose are then used for a research purpose. In this instance, further processing is allowed in terms of section 15(3) (*e*) of POPIA as the ‘*historical, statistical and research purpose*’ provision can be relied upon, provided that the ‘responsible party ensures that the further processing is carried out solely for such purposes and will not be published in an identifiable form’ (section 15(3)(*e*)). However, the inverse is not true: data collected initially for a research purpose, save for relying on one of the provisions described in section 15(3) of POPIA in the paragraph above, cannot simply be re-used for commercial purposes. So, *unless* the commercial purpose falls within one of the section 15(3)(*a*)–(*f*) provisions, it will be of an *incompatible purpose*. Save for these particular instances, POPIA restricts the repurposing of research data for commercial purposes.

Accordingly, in situations where the information is in the public domain or is deliberately made public by the data subject, in terms of section 15(3) (*b*) of POPIA, the information may be used for the further commercialised purpose. Moreover, and importantly, in terms of section 15(3)(*a*) of POPIA, where the consent of the data subject has been obtained, a researcher may use their information for a commercial purpose because POPIA deems it to then be compatible with the purpose of collection (in this case, for research). But this purpose (that is, commercialisation) would still need to be specific and explicitly defined – in line with section 13(1) of POPIA. Although Staunton et al.^40^ contended that POPIA can somehow be ‘purposively interpreted’ to allow for *broad* consent, we suggest that such an interpretation is incorrect.^41^ The language used in POPIA is unambiguous. Unless an exemption has been granted in terms of POPIA, the consent requirement in POPIA is *specific* consent – both for the initial collection (section 13(1)) and for the further processing (section 15(1) and (3)(*b*)).^42,43^

What then of commercial applications which have a wide (and undetermined) scope at the time of collection and repurposing? In this regard, a contract between the responsible party and the data subject may shed some light on whether, and under what conditions, further processing is allowable. If a research participant were to agree to donate their biological material and/or data for the purpose of *research*, and the contract was *silent or prevented* the research institution from subsequently using these data for commercial purposes, then the further processing of research data for commercial reasons would not be allowable (in terms of section 15(2)(*e*) of POPIA). Much will, however, depend on the initial and subsequent purpose (for research or for commercial use), what has been specifically consented to, and the nature and extent of such consent. As a solution of last resort, data subjects may need to re-consent if the stipulated consents are not in place.

### Enabling repurposed research data commercialisation

POPIA offers a further possible enabling solution – one that is an alternative to obtaining consent – in section 15(3)(*b*). This section deems information contained in a ‘public record’ or that which the data subject has ‘deliberately made public’ not incompatible with the purpose of collection. In addition to the research-related provisions already available to researchers, section 15(3)(*b*) of POPIA may prove helpful in supporting and growing science and technology innovations as envisaged in the South African National Development Plan.^44^ Such considerations inform economic growth and improve health systems, education, and infrastructure, by creating a public record of personal data and collaborating with participants to voluntarily make their information public.

By way of illustration, the Finnish biobank, Auria, demonstrates how biobank and personal research data can be used for commercial purposes to benefit participants.^45^ In this context, Finish citizens place trust in researchers and public institutions by willingly donating, and agreeing to make public, personal research data for commercial use.^46^ A model based on commercialisation – with reciprocal benefit and data made publicly available by the participant – that strengthens collaboration and participation with data subjects is advantageous in that it not only fosters long-term relationships between the parties (be they researchers or otherwise), but remains in compliance with data protection laws.^47^ Accordingly, and following POPIA, data collected for research purposes may be repurposed for commercial use if such data are contained in a public record or deliberately made public by the data subject. A model, not dissimilar to that of the one adopted in Finland, could be extended to South African research repositories that want to commercialise data.

## Conclusion and recommendations regarding the draft CCR

The draft CCR was developed, inter alia, to create legal certainty regarding the interpretation of POPIA’s provisions, to ensure that personal information used in research is protected, and to assist research institutions and independent researchers in complying with POPIA.^20^ POPIA does not mention the commercialisation of personal information, and therefore it is left up to the draft CCR to address this issue and guide researchers and research institutions, while protecting participants. But how does the draft CCR recognise, and manage, situations in which researchers want to use personal information, including special personal information, either (1) from a previous research project; or (2) for a different purpose (which could include commercial purposes)?^20^ Where researchers seek to use personal information collected from a previous research project for further research, or where researchers intend to use personal information for a new research-related purpose, the draft CCR requires researchers to provide certain information in a new research protocol, including: (1) the conditions under which the personal information was originally collected (including disclosures to research participants and information about consent); (2) how researchers will ensure that the personal information is used only for research and will not be published in an identifiable form; (3) how the notification requirement in section 18 of POPIA will be complied with; and (4) whether permission has been granted by the responsible party who initially processed the personal information.^20^ The draft CCR acknowledges the possibility of a changing purpose – which, although not explicitly stated in the draft CCR, may imply repurposing research data for commercial use. If this is indeed the case, this must be done in accordance with section 15(1) of POPIA.

However, the draft CCR concerns itself only with situations where personal information collected for another purpose is re-used for *research*.^20^ If the purpose changes to another type of research, it may be possible for this to fall within the scope of section 15(1) of POPIA as it remains within the boundaries of research and may be what the draft CCR envisioned when drafting this section. The draft CCR approaches research as a one-directional, inward flow of personal information collected for other purposes *into research*, but fails to provide for an outward flow of personal information initially collected for purposes of research *into commercialisation*. As we highlighted above, the commercialisation of research results is central to South African science and innovation policy – and a statutory duty to commercialise exists where public funds are used and commercially viable IP is involved.

Accordingly, given the importance of the commercialisation of research results to the South African research community, we suggest that the draft CCR address this important issue and lay out a *clear roadmap* for the South African research community on how to commercialise personal information initially collected for research. It would also be helpful for the draft CCR to include practical examples of consent statements for consent to the repurposing of personal data (initially collected for research) for commercialisation.

Against this recommendation, it can be argued that the topic of the commercialisation of research results is not research, and should therefore be excluded from the draft CCR. The draft CCR is, after all, intended to deal only with research. Although this argument would have technical merit, such a position is, in our estimation, myopic. Considerations about the commercialisation of personal data initially collected for research are often – and should be – a part of the design of research projects, and are a reality facing researchers and research institutions – whether initially planned or not. In other words, practically, decision-making regarding the commercialisation of research results is intrinsically integrated with research decision-making. Accordingly, the South African research community would benefit from a code of conduct for research that acknowledges this and considers how the commercialisation of research data might be realised.

## Significance:

For considerations of public policy, research in South Africa is not (merely) a purpose in itself, but a fundamental contributor to the South African economy. Because research data – which may consist of personal information – often has commercial value, it is important that we consider legal pathways for repurposing research data for commercial use. The POPIA draft Code of Conduct for Research should provide guidance in this regard.
